# P233: Incidence of ICU acquired nososcomial infections in University Hospital of Sahloul (Sousse-Tunisia)

**DOI:** 10.1186/2047-2994-2-S1-P233

**Published:** 2013-06-20

**Authors:** O Bouallègue, W Naija, H Said, Amel Nouria, Nadia Jaidane, Lamine Dhidah, Nourredine Boujaafar

**Affiliations:** 1 Microbiology Laboratory, Hospita of Sahloul, Sousse, Tunisia; 2 Surgical ICU, Sousse, Tunisia; 3 Hospital Hygiene Department, Hospital of Sahloul, Sousse, Tunisia

## Introduction

Health care-associated infections, or nosocomial infections are the most frequent adverse event in health-care delivery worldwide in particular in patients admitted to intensive care units because of the debilitated state of the patients and the sophisticated procedures.

## Objectives

The aim of the study is to present the preliminary result of the incidence study of nosocomial infection in ICU and to suggest some main solutions and perspectives for improvement.

## Methods

A prospective surveillance study was performed in the ICU at a university hospital of Sahloul in Sousse during the 6 months July through december 2010 to describe the epidemiologic profile of nosocomial infections.

## Results

A total of 47 patients (21.9%) were infected, and 13 (29.3%) had ICU-acquired infection. The infection incidence density was 20.7 per 1000 days. The most frequent types of ICU infection reported were: pneumonia (14.8%), followed by bloodstream infection (7.9%) and UTI (3.3%). Enterobacter cloacae species were the most frequent cause of UTI, Staphylococcus aureus was the most predominant in pneumonia, followed by Acinetobacter baumannii and Klebsiella pnaumoniae wich were the most frequently reported in bacteraemia. The overall mortality rates among infected and non-infected patients were 40,4% and 17,3% respectively.

## Conclusion

Many infection control measures, such as appropriate hand hygiene and the correct application of basic precautions during invasive procedures, are simple and low-cost, but require staff accountability and behavioural change, in addition to improving staff education and improving reporting and improving surveillance systems.

## Disclosure of interest

None declared

